# An unreported infraspinatus muscle variation—two-headed infraspinatus minor muscle and three-headed fusion with the teres minor muscle

**DOI:** 10.1007/s00276-022-02999-4

**Published:** 2022-08-12

**Authors:** Krzysztof Koptas, Nicol Zielinska, R. Shane Tubbs, Łukasz Olewnik

**Affiliations:** 1 Department of Anatomical Dissection and Donation, Medical University of Lodz, Lodz, Poland; 2 Department of Anatomical Sciences, St. George’s University, West Indies, Grenada; 3 Department of Neurosurgery, Tulane University School of Medicine, New Orleans, LA USA; 4 Department of Structural and Cellular Biology, Tulane University School of Medicine, New Orleans, LA USA; 5 Department of Surgery, Tulane University School of Medicine, New Orleans, LA USA; 6 Department of Neurosurgery, Ochsner Medical Center, New Orleans, LA USA

**Keywords:** Infraspinatus minor muscle, Rotator cuff, Shoulder anatomy, Infraspinatus muscle, Infraspinatus tendon, Anatomical variations

## Abstract

The infraspinatus muscle is situated under the scapular spine in the infraspinous fossa and inserts into the greater tuberosity of the humerus. It is a component of a crucial shoulder muscle group, the rotator cuff. There are a few interesting additional muscles in the infraspinal region. In the literature they are called the infraspinatus superficialis, infraspinatus minor and infraspinatus accessory muscles. The infraspinatus minor muscle is described as a superficial muscle bundle running under the scapular spine. During routine anatomical dissection, an unreported variation of the infraspinatus minor muscle was found. It derived from the inferior surface of the scapular spine and the infraspinous fossa. It had two heads. The superior head inserted on the greater tuberosity of the humerus. The inferior head inserted on the tendinous part of the infraspinatus muscle. There was also an unusual fusion of the infraspinatus muscle with the teres minor muscle. In this paper we will discuss the anatomical and physiological relationships of this morphological variation.

## Introduction

The infraspinatus is one of the rotator cuff muscles [17]. It is situated under the scapular spine in the infraspinous fossa. It is proximally attached to the dorsal scapular surface in the infraspinous fossa; its origin occupies the three-quarters of the fossa. The infraspinatus muscle inserts on the middle and lateral impression of the greater tuberosity [19]. It is perfused by the suprascapular and scapular circumflex arteries and is innervated by the suprascapular nerve.

The roles of the infraspinatus muscle are lateral rotation of the humerus. With three other muscles of the rotator cuff (subscapularis, supraspinatus, and teres minor) it maintains the humeral head in the glenoid fossa.

The shoulder girdle region is characterized by muscle variations. For example, the deltoid muscle can lack its acromial part [15]. It can create fusions with the pectoralis major, trapezius, infraspinatus or latissimus dorsi muscles [15]. The number of bellies of the subscapularis muscle ranges from one to nine. Accessory subscapularis muscles have been described in the literature. The infraspinatus muscle can be fused with the deltoid or teres minor muscles [15, 18]. Additional infraspinatus muscles such as the infraspinatus minor, infraspinatus superficialis and accessory infraspinatus have been found [14, 15].

Atrophy of the infraspinatus muscle is sometimes a reason why an athlete’s performance is limited [8, 9]. At the root of this pathology, compression of the suprascapular nerve in the spinoglenoid notch is common. Atrophy of the infraspinatus muscle restricts overhead throwing motions, which are important in sports, such as softball [16]

This case report describes a previously unreported variant of the infraspinatus muscle. The infraspinatus minor had two bellies with different distal attachments. The superior belly inserted into the greater tuberosity of the humerus near the infraspinatus muscle attachment. The inferior belly inserted into the tendon of the infraspinatus. There was also an unusual fusion with the teres minor muscle. We discuss the anatomical, clinical and physiological relationships of this morphological variation.

## Case report

**Fig. 1:**
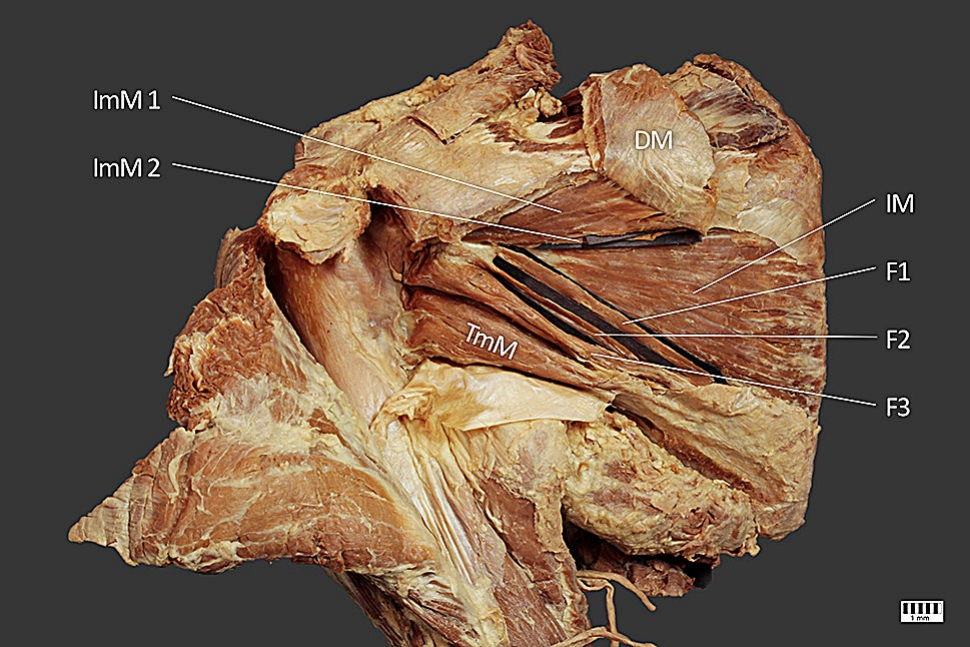
ImM1–superior head of the infraspinatus minor muscle; ImM2–inferior head of the infraspinatus minor muscle; IM–infraspinatus muscle; F1, F2, F3 – heads of the infraspinatus and teres minor muscles fusion; TmM–teres minor muscle; DM–deltoid muscle

The right upper limb from a female cadaver 83 years at death was subjected to routine anatomical dissection for research and teaching purposes in the Department of Anatomical Dissection and Donation, Medical University of Lodz, Poland. During this traditional anatomical dissection, we found a previously unreported variant of the infraspinatus muscle [20]. We identified a two-headed infraspinatus minor muscle and an infraspinatus muscle fused with the teres minor. The teres minor and infraspinatus muscles exchanged three muscle slips with each other (Fig. 1).

The infraspinatus muscle originated in the infraspinous fossa and inserted on the posterior surface of the greater tuberosity of the humerus. It comprised one muscle belly. It was fused with the teres minor muscle; the fusion was located at the beginnings of both muscles. There were also three muscle slips from the superior medial part of the teres minor muscle to the infraspinatus muscle (Fig. 1). The lengths of these bellies are given in Table 1.

**Table 1:** Lengths of distinct parts of the morphological variant represented by fusion between the teres minor muscle and the infraspinatus muscle

	Length (mm)
Superior	75.93
Medial	65.63
Inferior	89.21

**Fig. 2:**
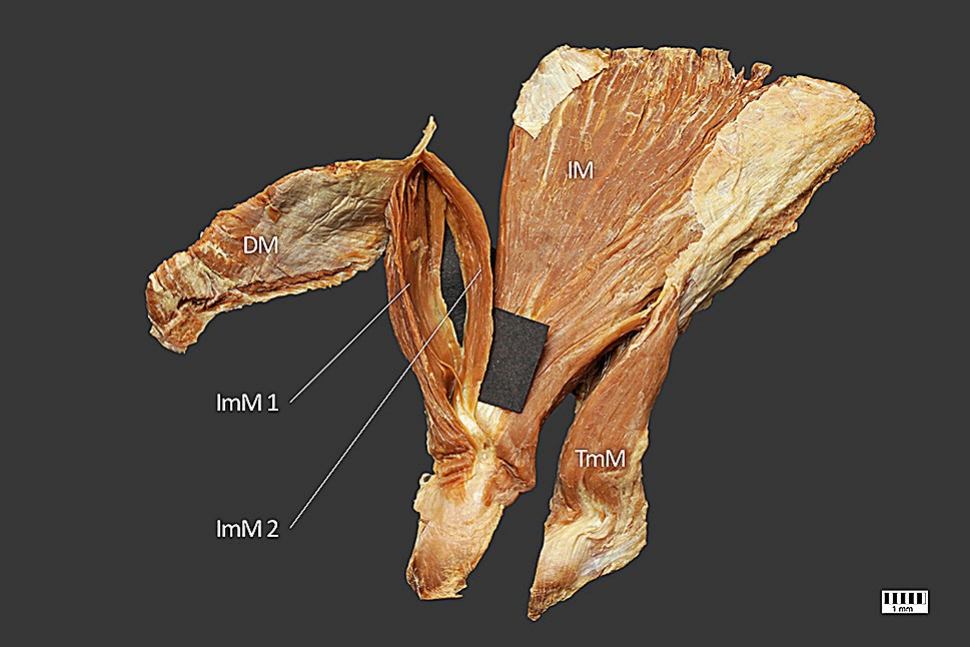
ImM1–superior head of the infraspinatus minor muscle; ImM2–inferior head of the infraspinatus minor muscle; IM – infraspinatus muscle; TmM–teres minor muscle; DM–deltoid muscle

**Fig. 3:**
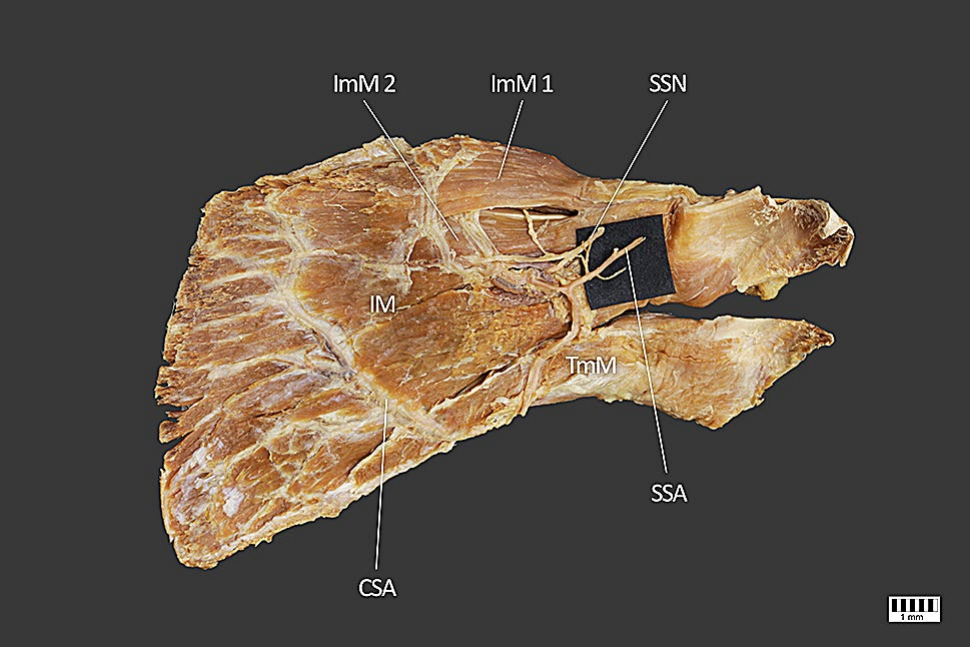
ImM1–superior head of the infraspinatus minor muscle; ImM2–inferior head of the infraspinatus minor muscle; IM–infraspinatus muscle; TmM–teres minor muscle; DM–deltoid muscle; SSA–suprascapular artery; CSA–circumflex scapular artery; SSN-suprascapular nerve

The infraspinatus minor muscle originated from the inferior surface of the scapular spine, and the medial part of the infraspinous fossa was also blended with the deltoid muscle fibers. The superior head ended on the greater tuberosity, and the inferior head ended on the tendinous part of the infraspinatus muscle (Fig. 2). The infraspinatus minor muscle was innervated by branches of the suprascapular nerve (Figs. 1, 3). Its morphometric measurements are given in Table 2.

**Table 2:** Morphometric measurements of the infraspinatus minor muscle

	Superior belly (mm)	Inferior belly (mm)
Length	77.12	44.14
Width PA	22.60	6.19
Thickness PA	1.61	1.08
Width DA	3.84	4.14
Thickness DA	0.43	0.48

*PA* proximal attachment, *DA* distal attachment

The infraspinatus and infraspinatus minor muscles were perfused by the suprascapular and scapular circumflex arteries (Fig. 3).

The muscles described above were very carefully dissected to minimize measurement errors. Then the prepared cadaver was subjected to detailed morphometric measurements and photographic documentation. The measurements were taken twice with up to 0.1 mm accuracy using an electronic caliper (Mitutoyo Corporation, Kawasaki-shi, Kanagawa, Japan). No other morphological variations were found.

## Discussion

During development, the deltoid, teres major, infraspinatus and supraspinatus muscles arise from a common premuscle mass continuous with the pectoral mass and the common arm sheath. In an 11 mm embryo the deltoid muscle has partially split off from the mass towards its origin from the acromion and clavicula. In embryos 14–16 mm in length it has much the adult form, with usually a distinct slip arising from the fascia over the infraspinatus muscle. In a 20 mm embryo it has practically the adult form and attachments. The development of the acromion from the cephalic border of the scapula partially separates the supraspinatus muscle from the infraspinatus in an 11 mm embryo. The infraspinatus and teres minor muscles are very closely associated from the outset and cover only a portion of the lateral surface of the scapula in an 11 mm embryo. In a 14 mm embryo the infraspinatus is quite distinct from the deltoid muscle, but does not cover the whole of the fossa infraspinata even in a 16 mm or 20 mm embryo [2–4].

Only a few variants of the infraspinatus muscle have been described previously. Macalister [15] described an infraspinatus muscle split into two laminae, which did not completely overlay each other Query[15]. The infraspinatus muscle fascia derived from the deltoid muscle to the infraspinatus muscle, and in the reverse direction from the infraspinatus to the deltoid [15]. A case found by Ashaolu et al. [1] showed two infraspinatus muscles attached to the medial surface of the infraspinous fossa and the humeral greater tuberosity [1]. There was a case that described an infraspinatus accessory muscle [14]. This additional muscle derived from the medial scapular border, ran directly under to the scapular spine and ended on the greater tuberosity of the humerus [14].

The following two variations are most significant for this paper. The infraspinatus muscle can be fused with teres minor [15, 18]. According to data completed by Mori [18], this fusion occurs in 10% of the Japanese population [18]. An additional muscle named the infraspinatus minor derives directly below the scapular spine and inserts into the greater tuberosity of the humerus. It can be observed not completely differentiated from the main muscle mass of the infraspinatus.

Kato et al. [13] redefined the structure of the infraspinatus muscle, dividing it into two parts, transverse and oblique. The oblique part has the shape of a fan. It originates from the infraspinous fossa and inserts into the greater tuberosity of the humerus. The transverse part originates from the inferior surface of the scapular spine and ends on the tendinous part of the oblique part of the infraspinatus muscle [13].

We identified the muscle masses located directly under the scapular spine not as the transverse part of the infraspinatus muscle, but as the infraspinatus minor muscle. We found that the muscle bundles were easy to separate from the main part of the infraspinatus.

Atrophy of the infraspinatus muscle has been observed by clinicians. The usual reason for this pathology is compression of the suprascapular nerve in the spinoglenoid notch. Infraspinatus muscle atrophy commonly occurs in sports with overhead throwing motions, such as tennis [8] and volleyball [9]. This pathology is not painful, but it limits the athlete’s achievements [8, 9]. Such atrophy can be treated with exercises that strengthen the external rotators of the glenohumeral joint. If the rehabilitation is not effective, this pathology can be treated surgically. Neurolysis of the compressed nerve succeeded by temporary immobilization and rehabilitation brings satisfactory results [9].

A double-headed infraspinatus minor muscle can provide additional strength and precision for movements in the shoulder girdle. It can be crucial for athletes practicing sports, such as softball, tennis or volleyball [8, 9, 16]. Fusion between the infraspinatus and teres minor muscles with three additional muscle slips can support both muscles. The infraspinatus minor muscle can take a supportive role in remplissage.

## Conclusions

The infraspinatus muscle can vary in many ways. It is important to know its possible variants. Each of them can change the biomechanics of the rotator cuff. Moreover, variants of this muscle should be cataloged so that if seen in future dissections or clinically, researchers will have a reference for this anatomy.

## Data Availability

Please contact authors for data requests (Łukasz Olewnik, PhD—email address: lukasz.olewnik@umed.lodz.pl).
